# Cerebrospinal Fluid B Cells Correlate with Early Brain Inflammation in Multiple Sclerosis

**DOI:** 10.1371/journal.pone.0002559

**Published:** 2008-07-02

**Authors:** Bettina Kuenz, Andreas Lutterotti, Rainer Ehling, Claudia Gneiss, Monika Haemmerle, Carolyn Rainer, Florian Deisenhammer, Michael Schocke, Thomas Berger, Markus Reindl

**Affiliations:** 1 Clinical Department of Neurology, Innsbruck Medical University, Innsbruck, Austria; 2 Department of Radiology I, Innsbruck Medical University, Innsbruck, Austria; Centre de Recherche Public-Santé, Luxembourg

## Abstract

**Background:**

There is accumulating evidence from immunological, pathological and therapeutic studies that B cells are key components in the pathophysiology of multiple sclerosis (MS).

**Methodology/Principal Findings:**

In this prospective study we have for the first time investigated the differences in the inflammatory response between relapsing and progressive MS by comparing cerebrospinal fluid (CSF) cell profiles from patients at the onset of the disease (clinically isolated syndrome, CIS), relapsing-remitting (RR) and chronic progressive (CP) MS by flow cytometry. As controls we have used patients with other neurological diseases. We have found a statistically significant accumulation of CSF mature B cells (CD19+CD138−) and plasma blasts (CD19+CD138+) in CIS and RRMS. Both B cell populations were, however, not significantly increased in CPMS. Further, this accumulation of B cells correlated with acute brain inflammation measured by magnetic resonance imaging and with inflammatory CSF parameters such as the number of CSF leukocytes, intrathecal immunoglobulin M and G synthesis and intrathecal production of matrix metalloproteinase (MMP)-9 and the B cell chemokine CxCL-13.

**Conclusions:**

Our data support an important role of CSF B cells in acute brain inflammation in CIS and RRMS.

## Introduction

Multiple sclerosis (MS), a chronic inflammatory demyelinating disease of the central nervous system (CNS), is generally assumed to result from an autoimmune attack directed against the myelin sheath [1]. The inflammatory reaction in the CNS of MS patients involves various components of the immune system, including T cells, B cells, macrophages but also antibodies and cytokines [2], [3].

Several key findings implicate a pathogenic role of B cells and antibodies in MS. Intrathecal immunoglobulin (Ig) synthesis and oligoclonal bands are found in more than 90% of MS patients [4], clonally expanded B cells accumulate in chronic MS lesions and in the CSF of MS patients [5]–[10], antibody-mediated demyelination was identified by histopathology [11], [12] and B cell targeted therapy has proven efficacy in phase II trials and open-label studies in MS [13]–[15].

B cells are found in the cerebrospinal fluid (CSF) during CNS inflammation, but are largely absent in non-inflammatory conditions [16]. In acute infectious or chronic inflammatory neurological diseases B cells may represent up to 30% of CSF cells [16]–[20]. In contrast to the periphery, the majority of CSF B cells are CD27 expressing antigen-experienced memory B cells [17], [20], [21].

Although these studies have clearly shown an important role of CSF B cells in CNS inflammation, their role in MS disease evolution and progression is less clear. New focal white matter lesions appear to develop following new waves of inflammation, involving immune cells which enter the brain from the peripheral blood and cause major blood brain barrier (BBB) leakage mediated by matrix metallproteinases (MMP). MMPs are supposed to be crucially involved in the degradation of the extracellular matrix and to facilitate subsequent leukocyte recruitment across capillary basement membranes to the site of inflammation [22], [23].

However, in contrast to early (relapsing-remitting) MS, the further inflammatory reaction in progressive MS seems to be compartmentalized within the CNS [24]. This is reflected by the formation of aberrant lymphatic tissue within the connective tissue compartments in the brain, the meninges and the perivascular spaces [25]. These ectopic follicular B cell structures might be crucial for a pathogenic and persisting B cell response driving disease progression [26], [27] and have been shown to contain B cells, T cells, plasma cells and a network of follicular dendritic cells producing homing lymphoid chemokines, such as CxCL-13 [25]. A recent study indicated that the intrathecal synthesis of CxCL-13 correlated with the number of CSF B cells [28].

We performed this prospective study to analyze CSF B cell phenotypes from patients at the onset of the disease (clinically isolated syndrome, CIS), relapsing-remitting (RR) and chronic progressive (CP) MS with the aim to investigate whether patients/subgroups of patients exhibit certain B cell phenotypes, whether different B cell subpopulations correlate with different MS disease courses and magnetic resonance imaging (MRI) characteristics, and whether we may identify patients for stratification for B cell focused treatment. In order to corroborate the involvement and biological activities of B cells in this study population we also analyzed MMP-9 and CxCL-13 in the CSF.

We report a statistically significant increase of CSF mature B cells (CD19+CD138−) and plasma blasts (CD19+CD138+) in CIS and RRMS. Further, this accumulation of B cells correlated with acute brain inflammation measured by MRI and with inflammatory CSF parameters such as the number of CSF leukocytes, intrathecal IgM and IgG synthesis and intrathecal detection of MMP-9 and CxCL-13.

## Methods

### Patients

MS patients and neurological controls were recruited prospectively from 2004 to 2008 at the Clinical Department of Neurology, Innsbruck Medical University, Austria. This study was approved by the ethical committee of Innsbruck Medical University (study Nr. UN2045, 217/4.12, 07.07.2004) and all patients gave written informed consent to the study protocol.

The following inclusion criteria had to be fulfilled by the group of CIS and MS patients: (1) clinical symptoms meeting revised “McDonald Criteria” [29], (2) positive MRI findings according to revised “McDonald Criteria” [29] and (3) presence of intrathecal immunoglobulin synthesis (elevated Ig-indices and/or oligoclonal IgG bands) [30]. 25 patients with a CIS, 20 patients with RRMS and 8 patients with CPMS (2 with secondary progressive-SP and 6 with primary progressive-PP MS) were included. All MS patients were diagnosed according to the established diagnostic criteria [29], [31] and a neurologist (A.L., R.E., C.G., T.B.) examined the patients, including assessment of the expanded disability status score (EDSS) and confirmation of relapse/disease progression during the follow-up period. 3 patients received high-dose corticosteroid treatment, whereas 3 patients received immunomodulatory treatments (2 patients received interferon-beta and one patient received intravenous immunoglobulins) at the time of lumbar puncture. 8 of 23 (31%) patients with a CIS converted to clinically definite MS during the follow-up period of 12 months. Demographic, clinical and CSF characteristics of all MS patients and neurological controls are shown in Table 1.

**Table 1:** Demographic, clinical and CSF data of analyzed patients.

	CIS	RRMS	CPMS	OND	p-value
n	25	20	8	30	
Females (%)	19 (76%)	16 (80%)	2 (25%) #	22 (73%)	0.02 2
Age (y) 1	26 (17–68) #	35 (15–63)	51 (36–63)	39 (21–74)	0.001 3
Duration (y) 1	0.1 (0.0–0.4)	4.9 (0.4–25.2)	7.8 (0.1–30.1)		<0.001 3
EDSS 1	2.0 (0.0–3.5)	2.0 (0.0–3.5)	5.8 (3.5–6.5)		0.001 3
Acute relapse	22 (88%)	14 (70%)	1 (13%)		<0.001 2
MRI Gd+ lesions	19 (76%) #	11 (55%) #	4 (50%) #	0/26 (0%)	<0.001 2
≥9 MRI T2 lesions	9 (36%) #	6 (30%) #	3 (38%) #	0/26 (0%)	0.03 2
OCB	24 (96%) #	18 (90%) #	7 (88%) #	4/27 (15%)	<0.001 2
CSF cells/µl 1	10 (1–58) #	7 (1–32) #	4 (2–7)	1 (0–211)	<0.001 3
IgG index 1	0.8 (0.5–3.1) #	0.9 (0.4–2.0) #	0.7 (0.4–1.7)	0.5 (0.4–1.5)	<0.001 3
IgM index 1	0.2 (0.0–0.8) #	0.2 (0.1–0.5)#	0.2 (0.0–0.8) #	0.1 (0.0–0.5)	<0.001 3
Q-Alb 1	4 (2–9)	5 (3–12)	7 (3–11)	5 (2–19)	ns 3
CxCL-13:					
CSF (pg/ml) 1	9 (1–468) #	8 (1–238) #	11 (1–94) #	1 (1–35)	<0.001 3
serum (pg/ml) 1	22 (1–177)	13 (1–80)	16 (1–104)	23 (1–302)	ns 3
ratio 1	0.6 (0.0–468.1) #	0.8 (0.0–238.4) #	1.3 (0.1–43.5) #	0.0 (0.0–4.3)	<0.001 3
MMP-9:					
CSF (ng/ml) 1	0.7 (0.0–21.4) #	0.5 (0.0–2.6) #	0.5 (0.0–0.8)	0.3 (0.0–99.8)	0.002 3
serum (ng/ml) 1	455 (180–1300)	435 (130–1798)	419 (223–777)	365 (79–1813)	ns 3
ratio (×1000) 1	1.6 (0.1–42.9) #	1.1 (0.0–7.8)	1.1 (0.1–3.5)	0.6 (0.0–396.9)	0.03 3

**1** data are shown as median (range), p-value: groups were compared using **2** Qui-Square test or **3** Kruskal-Wallis test and Dunn's multiple comparison post-hoc test, # statistically significant different from OND control group.

Abbreviations: n = number of patients, y = years, EDSS = expanded disability status score, acute relapse = number of patients with an acute relapse, MRI Gd+ lesions = presence of gadolinium-enhancing lesions on T1-weighted MRI (neg = negative, pos = positive), ≥9 T2 MRI lesions = ≥9 lesions on T2-weighted MRI, OCB = oligoclonal bands, Q-Alb = albumin quotient, MMP = matrix metalloproteinase, ratio = CSF∶serum ratio, ns = statistically not significant.

The control group consisted of 30 patients with other neurological diseases (OND). The OND group included 20 patients with noninflammatory OND (pseudotumor cerebri, migraine, psychogenic neurological symptoms, sinus venous thrombosis, cavernoma, vascular leukoencephalopathy, seizure, herniated vertebral disc, transient ischemic attack, spastic paraparesis, multiple system atrophy, neuropathic pain syndrome, cerebellar infarction, brain tumor, focal dystonia, ischemic transverse spinal cord syndrome) and 10 patients with inflammatory OND (myelitis, ventriculitis, viral meningoencephalitis, vasculitis, systemic lupus erythematosus, antiphospholipid syndrome, sarcoidosis and Guillain-Barré syndrome).

### Sample collection

CSF (3–8 ml), EDTA-treated whole blood and serum samples (Sarstaedt Monovettes, Nuembrecht, Germany) were obtained during standard diagnostic lumbar puncture and peripheral vein puncture, respectively. CSF samples were immediately analyzed for CSF cell populations within 30 minutes after lumbar puncture and cell-free supernatants were stored at −80°C for further diagnostic and scientific purposes. Serum was prepared by centrifugation, 10 min at 3000 rpm, and stored at −20°C until analysis was performed.

CSF samples were routinely analyzed for cell counts, quantitative (IgM and IgG indices) and qualitative (oligoclonal bands) analysis of intrathecal Ig-synthesis, and CSF to serum albumin ratio (albumin-quotient) as indicators of the blood-brain barrier status immediately after lumbar puncture using standard methods (Table 1).

### Characterization of CSF cell populations by flow cytometry

Staining of CSF cells was done as recently described [16] with the following modifications: CSF was immediately spun down after lumbar puncture for 10 minutes at 1500 rpm. After removing the supernatant, pellets were resuspended in 50 µl BD Cell-Wash (BD Biosciences, San Jose, CA, USA) and CSF cells were stained with fluorochrome-labeled antibodies to the following human leukocyte surface antigens (all BD Biosciences) for a total of 30 minutes at room temperature in the dark:

1. *Mature B cells (CD19+CD138−), plasma blasts (CD19+CD138+) and plasma cells (CD19-CD138+)*: 5 µl CD-45 PerCP (BD 345809) 10 µl CD19-FITC (BD 245776) and 10 µl CD138-PE (BD 347192).
2. *B cells (CD19+) and T cells (CD3+)*: 10 µl TriTest CD45-PerCP/CD3-FITC/CD19-PE (BD 342412).

Only when enough CSF cells were available the following stainings were also included:

3. *Monocytes*: 5 µl CD-45 PerCP (BD 345809) 5 µl CD14-FITC (BD 345784) and 5 µl HLA-DR-PE (BD 340689).
4. *Natural killer cells*: 10 µl TriTest CD45-PerCP/CD3-FITC/CD16+56-PE (BD 342411).
5. *Memory B cells*: 5 µl CD-45 PerCP (BD 345809) 10 µl CD27-FITC (BD 555440) and 10 µl CD19-PE (BD 345777).

Erythrocytes were lysed for 10 minutes using 2 ml of lysing solution (BD Biosciences) according to manufacturer's protocol. Then, the tubes were centrifuged and the supernatants discarded. After one additional washing step with 2 ml BD Cell-Wash, CSF lymphocyte subpopulations were analyzed using three-color flow cytometry on a BD FACScan with Cell Quest software (BD Biosciences). Lymphocytes and monocytes were gated according to their forward (FSC) and side scatter (SSC) properties. A minimum number of 1000 events for each CSF staining was acquired for analysis with lyse-wash instrument settings, threshold FL-3 (PerCP) channel at 300 and the gate adjusted SSC versus FL-3. Cell populations are shown in % of total lymphocytes.

### Determination of CSF and serum levels of MMP-9 and CxCL-13

CSF and serum concentrations of matrix metalloproteinase (MMP)-9 (human MMP-9 DuoSet, DY911, R&D Systems, Minneapolis, USA) and CxCL-13 (DuoSet human CXCL13/BLC/BCA-1, Catalog No. DY801, R&D Systems, Minneapolis, USA) were measured with commercially available enzyme-linked immunosorbent assays (ELISA) according to manufacturer's protocol. CSF samples were analyzed at a dilution of 1∶51 (for MMP-9) and undiluted (for CxCL-13). Serum samples were analyzed at a dilution of 1∶1501 (for MMP-9) and 1∶3 (for CxCL-13). In order to correct for a passive transfer of MMP-9 and CxCL-13 we calculated a ratio using the following formula: MMP-9 ratio = MMP-9 _CSF_/MMP-9 _Serum_.

### MRI protocol

All MS patients and 26 of 30 OND patients were examined by a standardized brain MRI protocol (duration about 18 min) at the time of diagnostic lumbar puncture. Patients were scanned on a 1.5 T whole-body MR scanner (Magnetom Avanto, Siemens, Germany) using the following protocol:

1. Coronal MPRage (repetition time (TR) 1600 ms, Inversion time (TI) 800 ms, echotime (TE) 3.44 ms, matrix 256×224, field of view 230×193 mm, slice thickness of 1.2 mm, number of excitations 1, iPAT factor 2)
2. Transversal diffusion tensor imaging by using a EPI sequence (TR 6000 ms, TE 94 ms, matrix 128×128 interpolated to 256×256, field of view 220×220 mm, slice thickness of 3 mm, 35 slices, iPAT factor 2, diffusion-sensitizing gradients in six directions)
3. Sagittal SPACE 3D with darkfluid preparation (TR 6000 ms, TI 2200 ms, TE 328 ms, matrix 256×236, field of view 240×221 mm, slice thickness 0.9 mm)
4. Pause of at least five minutes after administration of contrast agent (Gadolinium-DTPA)
5. Repetition of sequence 1

### Statistical analysis

Statistical analysis (means, medians, range, standard deviations), significance of group differences and linear regression were evaluated using SPSS software (release 14.0, SPSS Inc., USA) and GraphPad Prism 5 (GraphPad, San Diego, USA). The distribution of groups was determined via Kolmogorov-Smirnov test. Between-group comparisons were performed with Kruskal-Wallis test, Dunn's multiple comparison post-hoc test, Mann-Whitney *U* test, Fisher's exact test and Qui-square test. Correlation of parameters was analyzed with Spearman's non-parametric correlation. Statistical significance was defined as two-sided p-value<0.05 and Bonferroni corrections were made for multiple comparisons when appropriate.

## Results

### The frequency/percentage of CSF B cells is increased in acute CNS inflammation

**Figure 1:**
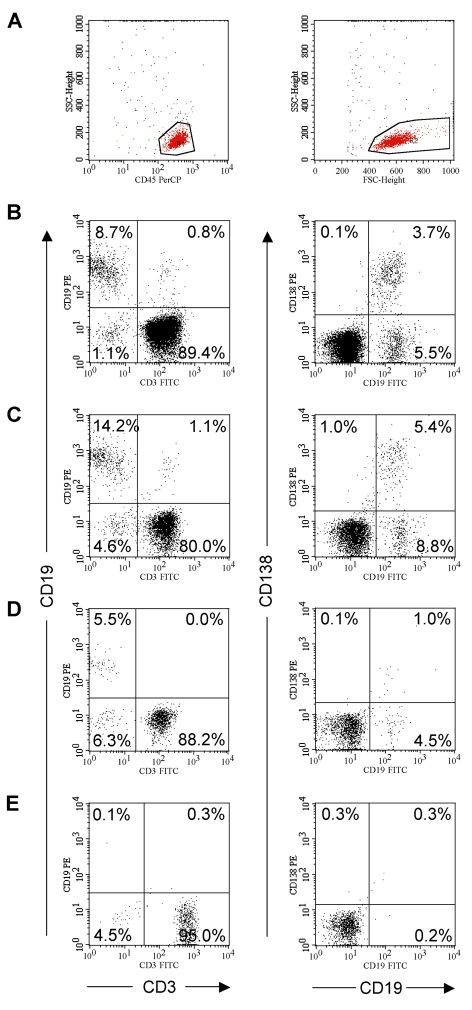
CSF B cell subsets. (A) Dot plots of CSF leucocytes according to their CD45-PerCP versus side scatter properties (left panel) and forward versus side scatter properties (right panel). Region 1 (R1, left panel) was used for acquisition of a minimum number of 1000 events and region 2 (R2, right panel) was used for analysis. CSF analysis of representative patients with a CIS (B), RRMS (C), CPMS (D) and OND (E) for the presence of CD3+ T cells and CD19+ B cells (left panel), and CD19+CD138− mature B cells, CD19+CD138+ plasma blasts and CD19−CD138+ plasma cells (right panel). The numbers represent the relative percentages of these cell populations.

CSF samples were analyzed for the presence of B cells by flow cytometry. During acquisition, lymphocytes were gated according to CD45-PerCP staining and SSC properties and subsequent analysis was done using their FSC versus SSC properties (Figure 1A).

The percentage of T cells and B cells within CSF was measured in each specimen using monoclonal antibodies to CD3 and CD19. Whereas T cells were present in all samples, B cells were barely detectable in the CSF of OND patients, but present in almost all patients with CIS, RRMS and CPMS (Figure 1B-E). CSF B cells were then characterized by a combination of CD19 and CD138 stainings (Figure 1B–E). While the vast majority of all CSF B cells were CD19+CD138− mature B cells (range 0–12.4%) and CD19+CD138+ (range 0–7.2%) plasma blasts, only a scant number of CSF cells had the CD19−CD138+ phenotype of long-lived plasma cells (range 0–1.3%). Additional stainings, which were however only possible in a few MS patients (due to limited CSF amounts), indicated that the majority of CSF B cells belong to the CD19+CD27+ memory B cell subset, while only a minority are naïve CD19+CD27− B cells (data not shown). In consistence with previous reports [16], [17], [20] we never found CD19+CD138+ plasma blasts in the peripheral blood of MS patients (data not shown).

**Figure 2:**
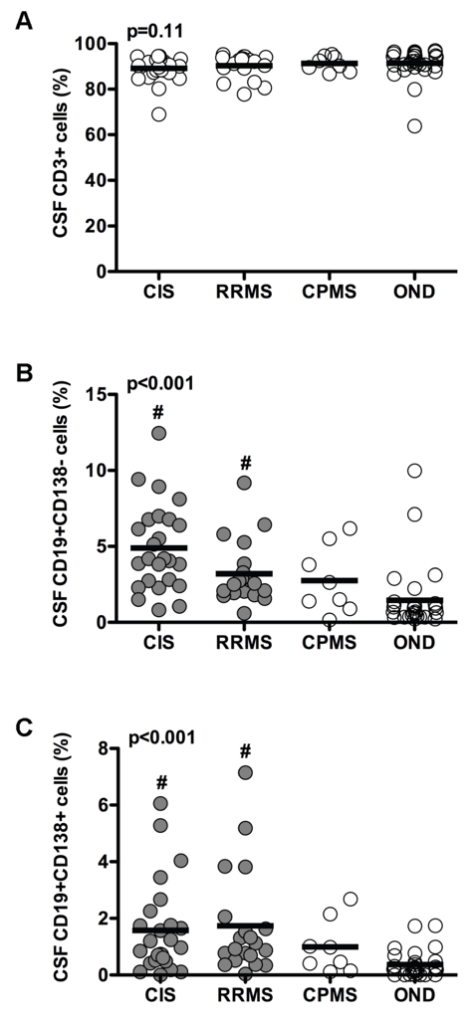
Percentages of CD3+ T cells (A), CD19+CD138− mature B cells (B) and CD19+CD138+ plasma blasts (C) in the CSF of patients with a CIS, RRMS, CPMS and OND. Individual data points are shown as circles and horizontal bars indicate means. Data were compared using the Kruskal-Wallis test and Dunn's multiple comparison post-hoc test and overall p-values are shown in each figure. # = significant differences to the OND group.

From Figure 2A it is evident that most of the lymphocytes present in CSF samples are T cells (range 63.8–96.9%), but without significant differences between different MS disease courses and OND (p = 0.11). In contrast, CSF CD19+CD138− mature B cells and CD19+CD138+ plasma blasts were significantly increased in CIS and RRMS as compared to OND (p<0.001, Figure 2 B and C). Neither mature B cells nor plasma blasts were significantly increased in CPMS. Within OND patients CSF CD19+CD138− mature B cells and CD19+CD138+ plasma blasts were only detectable in some patients with inflammatory OND (myelitis and systemic lupus erythematosus).

As mentioned above CD19−CD138+ long-lived plasma cells were nearly absent in all disease groups and no significant differences were found between groups. Further, we found no statistical significances in the frequencies of natural killer cells (n = 83) and monocytes (n = 46) between the groups analyzed.

Within MS patients CSF mature B cell, but not plasma blast, counts were significantly higher in CIS and RRMS than in CPMS (p = 0.028). Within CPMS, patients with SPMS (n = 2) had higher CSF CD19+CD138− and CD19+CD138+ counts than PPMS (n = 6, p = 0.046). Further, CSF CD19+CD138− mature B cells were significantly increased (p = 0.01) in patients with an acute relapse (median 3.9, range 1.1–12.4%) as compared to patients in remission (median 2.3, range 0.6–6.8%) and progression (median 1.5, range 0.1–6.2%). No significant differences were found for T cells, plasma blasts and other CSF cell populations. CSF T cells, mature B cells and plasma blasts did not significantly correlate with other clinical parameters such as age, gender, disease duration, EDSS and conversion to clinically definite MS.

### CSF B cells correlate with paraclinical markers of CNS inflammation

We next analyzed whether CSF cell populations correlate with MRI activity, defined by the number of lesions on T2-weighted MRI (2 groups: <9 and ≥9 T2 lesions) and the presence of Gd-enhancing lesions on T1-weighted MRI (2 groups: Gd-enhancing lesions present or not) at the time point of CSF sampling. As shown in Table 2, CSF mature B cells and plasma blasts were highly significantly associated with a higher disease activity on MRI measured by the presence of numerous (≥9) T2 lesions and the presence of Gd-enhancing lesions, indicating an acute inflammatory reaction within the CNS and concomitant blood-brain barrier dysfunction. This association with MRI parameters was also seen for higher total numbers of CSF cells with the presence of Gd-enhancing lesions (median 8.5, range 2.3–58.0 versus median 2.7, range 0.3–210.7 cells/µl, p<0.001), but not for MRI T2 lesions. CSF T cells, NK cells and monocytes were, however, not correlated with MRI parameters.

**Table 2:** Correlation of CSF B cells with other inflammatory parameters.

	CD19+CD138− mature B cells	CD19+CD138+ plasma blasts
MRI Gd lesions:	p<0.001 2	p<0.001 2
MRI Gd neg 1	1.1 (0.1–12.4)	0.3 (0.0–5.2)
MRI Gd pos 1	3.3 (0.6–9.4)	1.1 (0.0–7.1)
MRI T2 lesions:	P<0.001 2	p = 0.009 2
<9 lesions 1:	1.1 (0.1–12.4)	0.3 (0.0–5.2)
≥9 lesions 1:	3.3 (0.6–9.4)	1.1 (0.0–7.1)
CSF CD19+CD138− cells		R = 0.650, p<0.001 3
CSF CD19+CD138+ cells	R = 0.650, p<0.001 3	
CSF CD3+ cells	R = −0.561, p<0.001 3	R = −0.395, p<0.001 3
CSF CD56+ cells	R = −0.275, ns	R = −0.199, ns
CSF CD14+ cells	R = −0.150, ns	R = −0.209, ns
CSF cells/µl	R = 0.550, p<0.001 3	R = 0.443, p<0.001 3
Q-Alb	R = −0.136, ns	R = −0.052, ns
IgM index	R = 0.488, p<0.001 3	R = 0.585, p<0.001 3
IgG index	R = 0.515, p<0.001 3	R = 0.548, p<0.001 3
OCB:	p<0.001 2	p<0.001 2
OCB neg 1	0.7 (0.2–9.4)	0.2 (0.0–1.7)
OCB pos 1	2.9 (0.1–12.4)	1.0 (0.0–7.1)
CxCL-13:		
CSF (pg/ml)	R = 0.574, p<0.001 3	R = 0.522, p<0.001 3
serum (pg/ml)	R = −0.037, ns	R = −0.178, ns
ratio	R = 0.395, p<0.001 3	R = 0.434, p<0.001
MMP-9:		
CSF (ng/ml)	R = 0.415, p<0.001 3	R = 0.440, p<0.001 3
serum (ng/ml)	R = 0.206, ns 3	R = 0.144, ns 3
ratio	R = 0.262, p = 0.01 3	R = 0.310, p = 0.005 3

**1** data are shown as median (range), p-value: groups were compared using **2** Mann-Whitney U test or **3** Spearmans nonparametric correlations.

Abbreviations: MRI Gd+ lesions = presence of gadolinium-enhancing lesions on T1-weighted MRI (neg = negative, pos = positive), MRI T2 lesions = number of lesions on T2-weighted MRI, Q-Alb = albumin quotient, OCB = oligoclonal bands, MMP = matrix metalloproteinase, R = Spearman's correlation coefficient, ns = statistically not significant.

CSF mature B cells and plasma blasts were also highly significantly correlated with other inflammatory CSF parameters, such as CSF leukocyte counts and intrathecal IgM and IgG synthesis measured by IgM and IgG indices and positive oligoclonal bands (Table 2). Further, both CSF mature B cells and plasma blasts were negatively correlated with CSF CD3+ cells, whereas we found no significant correlation with CSF CD56+ NK cells and CD14+ monocytes.

From Table 1 it is evident that CSF, but not serum, CxCL-13 levels were significantly increased in CIS, RRMS and CPMS. Further, CSF and serum CxCL-13 levels of patients with CIS, RRMS and CPMS had similar ranges and also CSF∶serum ratios were significantly increased in these patients. Both, CSF CxCL-13 levels and CxCL-13 CSF∶serum ratios showed a highly significant correlation with CSF mature B cells and plasma blasts (Table 2).

From Table 1 it is also evident that CSF levels of MMP-9, but not serum levels, were significantly increased in CIS and RRMS, and the CSF∶serum ratio was also increased in CIS. Further, CSF MMP-9 levels were higher (p = 0.04) in patients with an acute relapse (median 0.6, range 0.0–5.7 ng/ml) than in remission (median 0.3, range 0.1–1.5 ng/ml) or progression (median 0.5, range 0.0–0.8 ng/ml). CSF MMP-9 levels (median 0.3, range 0.0–1.0 versus median 0.6, range 0.0–5.2 ng/ml, p<0.001) and CSF∶serum ratios (median 0.6, range 0.0–2.5 versus median 1.6, range 0.0–31.6, p<0.001) were also significantly increased in patients with Gd-enhancing MRI lesions and showed a highly significant correlation with CSF mature B cells and plasma blasts (Table 2). This significant correlation of CSF MMP-9 levels (R = 0.622, p<0.001) and CSF∶serum ratios (R = 0.483, p<0.001) was also seen for the total number of CSF cells, but not for CSF T cells, NK cells and monocytes.

## Discussion

Our prospective study was designed to compare the B cell inflammatory response between relapsing and progressive MS by characterizing the CSF cell profiles from patients at the onset of the disease (CIS), RRMS, CPMS and OND. Our data clearly indicate that the cellular B cell response, as measured in the CSF, is clearly more pronounced in acute inflammation, i.e. CIS and RRMS, than in CPMS and OND. The majority of these B cells showed the expression pattern of mature B cells (phenotype: CD19+CD138−), which have the ability to remain in the CNS compartment for years [17]. We have also observed an increased frequency of CD19+CD138+ plasma blasts in CIS and RRMS, but not in CPMS and OND. In contrast, only a scant amount of long-lived plasma cells (phenotype: CD19−CD138+) was detected in the CSF. Consistently, a predominance of mature B cells and short-lived plasma blasts in CSF of patients with MS and acute neuroinfection has been reported previously [16]–[19], [21]. By contrast, a recent report also showed increased number of CD19−CD138+ plasma cells in the CSF of patients with neuroinflammatory diseases [20].

Our finding that CSF B cells are correlated with other paraclinical markers of acute brain inflammation such as high numbers of MRI T2 lesions, the presence of Gd-enhancing MRI lesions, total number of CSF cells, intrathecal IgM and IgG synthesis and intrathecal MMP-9 and CxCL-13 production indicate a direct involvement of B cells in acute brain inflammation. These findings might also explain some of the dramatic effects seen in recent therapeutic trials in RRMS patients. Natalizumab, a recently re-approved drug for MS treatment, has been shown to reduce the numbers of CD19+ B cells and CD138+ plasma cells within the CSF [32] which correlated with the reduction of relapse rates as well as of the inflammatory disease activity on MRI. Similar effects were observed with rituximab, a monoclonal antibody selectively depleting CD20+ B cells [13]–[15], thus indicating an influence of B cells on disease pathogenesis and ongoing immune responses. Although B cell targeted therapy has proven efficacy in RRMS patients and clinical trials in CPMS patients are ongoing, we do not yet know which patients could most probably benefit from a B cell targeted therapy. It is thus tempting to speculate whether the CSF B cell profile might be helpful in identifying patients that might benefit from these treatments.

Despite the difference seen in the frequency of CSF B cells between RRMS and CPMS, humoral factors like IgM, IgG and CxCL-13 were found at increased levels in all MS subgroups thus raising the question on their source. Recent studies indicated that the continuous maturation of memory B cells into plasma blasts, driven by disease-associated antigens, might proceed in meningeal germinal centre-like structures within the CNS [25], [27], [33]. Since the ectopic expression of CxCL-13 is considered to promote the formation of lymphoid-like structures [34] its expression in MS lesion might have similar effects in this disease [25], [35]. Further, in a recent study a strong correlation between CxCL-13 levels and inflammatory activity in MS was observed, which was reflected by an upregulation of CxCL-13 in actively demyelinating MS lesions and increased CxCL-13 in the CSF [28]. Thus the chemokine CxCL-13 might play a critical role in organizing immune responses within CNS by providing favorable microenvironmental preconditions for compartmentalizing B cell response and sustaining a chronic inflammatory process in the CNS. The lower frequencies of mature B cells and plasma blasts in CPMS, as well as the absence of CD19-CD138+ long-lived plasma cells in the CSF observed in our study and by others [16], [17], could thus reflect the retaining of this cell subset within germinal centers in the meninges.

In our study we found a significant correlation of CSF MMP-9 levels and CSF∶serum ratios with the number of mature B cells and plasma blasts, but not with any other cell type. Further, CSF MMP-9 levels were increased in CIS, in patients with an acute relapse and in patients with Gd-enhancing MRI lesions thus confirming previous results [36], [37]. Our finding that the correlations between CSF cell populations and intrathecal MMP-9 production were restricted to B cell subsets may either indicate that B cells are responsible for the release of MMP-9 [38], [39] or that the MMP-9 release facilitates, amongst other molecules, B cell migration through the BBB.

In conclusion the results of our study reveal an accumulation of mature B cells and plasma blasts in the CSF of patients with a CIS and RRMS, which is associated with an increased intrathecal CSF cell count and MMP-9, CxCL-13, IgG and IgM synthesis. Although the specificity of these intrathecal B cells is not known and the role of antibodies in the immunopathogenesis of MS is still unclear, the findings of this study confirm a role of B cells and humoral factors in MS disease activity. Because the core phase of our study has been scheduled for a maximum of 3 years at study initiation, an extension phase is now planned in order to collect follow-up clinical data of the study cohort (progression index, time to conversion to clinically definite and chronic-progressive MS). These studies will hopefully clarify the implications of different CSF cell subsets for prognosis and potentially add information to the decision process of therapeutic strategies in MS.
